# Spatiotemporal Visualization of Photogenerated Carriers on an Avalanche Photodiode Surface Using Ultrafast Scanning Electron Microscopy

**DOI:** 10.3390/nano14030310

**Published:** 2024-02-03

**Authors:** Yuan Tian, Dong Yang, Yu Ma, Zhongwen Li, Jun Li, Zhen Deng, Huanfang Tian, Huaixin Yang, Shuaishuai Sun, Jianqi Li

**Affiliations:** 1 Beijing National Laboratory for Condensed Matter Physics, Institute of Physics, Chinese Academy of Sciences, Beijing 100190, China; tyan@iphy.ac.cn (Y.T.); dyang@iphy.ac.cn (D.Y.); mayu@iphy.ac.cn (Y.M.); lizhongwen@iphy.ac.cn (Z.L.); junli@iphy.ac.cn (J.L.); zhen.deng@iphy.ac.cn (Z.D.); hftian@iphy.ac.cn (H.T.); hxyang@iphy.ac.cn (H.Y.); sss@iphy.ac.cn (S.S.); 2 School of Physical Sciences, University of Chinese Academy of Sciences, Beijing 100049, China; 3 Songshan Lake Materials Laboratory, Dongguan 523808, China

**Keywords:** photogenerated carrier, ultrafast scanning electron microscopy, avalanche photodiode (APD), carrier transport, surface photovoltage

## Abstract

The spatiotemporal evolution of photogenerated charge carriers on surfaces and at interfaces of photoactive materials is an important issue for understanding fundamental physical processes in optoelectronic devices and advanced materials. Conventional optical probe-based microscopes that provide indirect information about the dynamic behavior of photogenerated carriers are inherently limited by their poor spatial resolution and large penetration depth. Herein, we develop an ultrafast scanning electron microscope (USEM) with a planar emitter. The photoelectrons per pulse in this USEM can be two orders of magnitude higher than that of a tip emitter, allowing the capture of high-resolution spatiotemporal images. We used the contrast change of the USEM to examine the dynamic nature of surface carriers in an InGaAs/InP avalanche photodiode (APD) after femtosecond laser excitation. It was observed that the photogenerated carriers showed notable longitudinal drift, lateral diffusion, and carrier recombination associated with the presence of photovoltaic potential at the surface. This work demonstrates an in situ multiphysics USEM platform with the capability to stroboscopically record carrier dynamics in space and time.

## 1. Introduction

The excitation, transport, and recombination of charge carriers in semiconductor materials determine their viability for numerous applications in photocatalytic and optoelectronic devices like photodiodes, photodetectors, and solar cells [1,2,3]. With the development of functional devices that follow the trend of miniaturization, there is an increasing desire to understand the behavior of photoexcited carriers on surfaces and at interfaces [4,5,6]. Among various types of semiconductor optoelectronic devices, avalanche photodiodes (APDs) are characterized by the multiplication of electron-hole pairs generated by the absorption of incident photons and play important roles in a variety of applications including optical communications, three-dimensional lidar detection, and photoluminescence [7,8,9,10]. In particular, separated absorption, grading, charge, and multiplication (SAGCM)-structure InGaAs/InP APDs have attracted widespread attention because of their high sensitivity, fast response, large internal gain, and low power consumption [11,12,13,14]. However, most studies on InGaAs/InP APDs have focused on their structural design and fabrication for the purpose of performance improvement; the non-equilibrium dynamics of photogenerated carriers on the device surface have seldom been discussed. In comparison to stroboscopic optical techniques [15,16,17,18,19] of which spatial resolution is constricted laterally by the diffraction limitation (>100 nm) and longitudinally by the penetration depth, time-resolved ultrafast scanning electron microscopy (USEM) combines the nanometer spatial resolution of traditional scanning electron microscopy (SEM) [20] and the sub-picosecond temporal resolution provided by photoemission pulsed electrons [21,22,23,24]. In USEM, similar to ultrafast electron microscopy in transmission mode [25,26], a pulsed pump laser beam from a femtosecond laser system is directed onto the specimen and excites it into nonequilibrium states, while in contrast, USEM collects the signal of secondary electrons (SEs), which originate from inelastic interactions between the primary photoemission electron pulses and the specimen in the near-surface region, producing snapshot SEM images at certain time points before or after laser excitation via raster scanning. After being excited by the optical pulse, the spatial distribution and energy-band state of surface carriers change considerably on an ultrashort time scale [27]. Upon scattering by the pulsed primary electrons, SEs ejected from a very thin surface layer of the order of a few nanometers thick characterize the behavior of surface carriers in real time, making this signal exclusively sensitive to surface carrier dynamics. Spatial charge-carrier contrast on a surface is the unique imaging strategy of USEM.

The application and development of USEM requires bright, stable, and cost-effective photoemission electron sources. Current USEM setups generally use Schottky field emitters. To generate pulsed photoelectrons, the filament current of the field emitter is turned off to suppress continuous electrons, and a periodic flash process is usually required to prevent contamination of the emission tip and fluctuations of the cathode work function. Ultrahigh vacuum conditions are needed for field emission and even higher for photoemission, which increases cost. The number of photoelectrons per pulse from a field emitter is typically less than one, which makes the signal-to-noise ratio of SEM images extremely low. An alternative is to use large-diameter planar emitters that can photogenerate sufficient probe current reliably within a reasonable acquisition time.

Here, we demonstrate the first USEM setup equipped with a LaB_6_ thermionic emission source, which simultaneously achieves SEM spatial resolution together with the temporal resolution of the optical pump–electron probe technique, enabling the visualization of transient carrier dynamics with a variety of critical times. By integrating a femtosecond laser into an SEM setup, pulsed photoelectrons are generated for ultrafast imaging with little sacrifice in spatial resolution. By obtaining the spatiotemporal evolution of SEM contrast on the surface of an InGaAs/InP APD device, the surface photovoltage (SPV) effect [28] and the entire spatiotemporal processes of carrier drift, diffusion, and recombination are able to be studied.

## 2. Materials and Methods

### 2.1. Instruments

**Figure 1:**
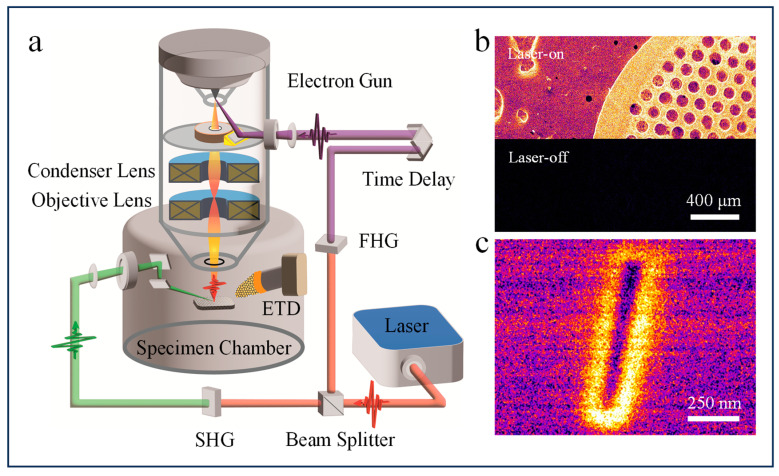
Conceptual scheme of the ultrafast scanning electron microscopy (USEM) and its performance in photoemission mode. (**a**) Schematic illustration of the USEM setup. The femtosecond laser is integrated with SEM by generating a pulsed photoemission electron beam and inducing surface dynamics in a specimen. A time delay line is used to control the arrival time of the pulsed electrons at the specimen surface relative to that of the pump laser. (**b**) USEM image of a copper grid captured in photoemission mode (upper) and with the electron-generating laser switched off (lower). (**c**) USEM image of a trench fabricated by a focused ion beam in a silicon substrate. It indicates that the spatial resolution of photoemission mode is approximately 20 nm.

Our home-built USEM setup integrated a femtosecond laser (FemtoYL, YSL Photonics Inc., Wuhan, China) with a commercial scanning electron microscope (EM6900, KYKY Inc., Beijing, China) equipped with a thermionic emission gun, as shown in Figure 1a. The fundamental pulsed laser beam with a center wavelength of 1030 nm, repetition rate of 5 MHz, and temporal pulse duration of 300 fs was split into two laser beams using a beam splitter, both of which were focused onto nonlinear crystals for fourth-harmonic generation (FHG) and second-harmonic generation (SHG), respectively. The former (257 nm) passed through a convex lens with a focal length of 200 mm and an entrance window of CaF_2_ glass. After being reflected by an ultraviolet (UV)-enhanced aluminum mirror, it converged onto the thermionic LaB_6_ cathode tip at an incident angle of 30°, thereby generating primary photoelectrons. Different from the traditional side-illumination method for wire-like field emitters [29,30,31] or backside illumination for flat photocathodes [32,33,34], a planar LaB_6_ cathode with a diameter of 100 µm, which is consistent with the size of the electron-generating light spot, means that this bottom-up photoelectron generation scheme is appropriate. In continuous emission mode, the vacuum required for the LaB_6_ cathode is generally much lower than that for field emitters. However, in pulsed photoemission mode, the poor vacuum can cause rapid contamination of the photocathode surface and reduce emission efficiency. Therefore, we added a getter ion pump to improve the vacuum of the thermionic gun to ~10^−6^ Pa. A long-term stable plateau of about 3 h and high photoelectron numbers in photoemission mode facilitated the time-resolved image acquisition process with a longer flash period and fewer repetition periods. The other frequency-doubled laser branch (515 nm) for pumping entered the modified specimen chamber horizontally, and its direction was changed by two reflectors placed face-to-face on an extension arm fixed to the chamber wall. The reflectors were positioned far enough apart to barely distort the electric field distribution around the specimen stage. The pump laser was focused into an elliptical spot on the specimen surface at an incident angle of 60° to the SEM electron optical axis. Our USEM setup allows the addition of multiple conditions (heat, cool, strain, twist, compression, etc.) to the sample in situ because of its compatibility with sample holders used in transmission electron microscopes. SEs scattered by primary photoelectron pulses, whether from the laser pump or unpumped region, were collected by a positively biased Everhart–Thornley detector (ETD). A frequently encountered obstacle in USEM research is that the pump laser not only initiates dynamics but is also scattered by the sample or components in the chamber (pole piece, sample stage, etc.). This means that the images become saturated even though only a small fraction of the photons eventually reach the photomultiplier tube (PMT) of the ETD. To prevent stray photons from entering the detector while ensuring that enough electrons are present for imaging, we retrofitted the SE detector by completely wrapping the light guide in front of the PMT and covering the surface of the cathodoluminescent scintillator facing the specimen with an aluminum coating with a thickness of 200 nm. A digital delay line located in the UV light path was used to control the time interval between the electron pulse and pump pulse while the spatiotemporal SEM contrast variation was recorded simultaneously.

The filament current was set to 1.3 A so that thermionic emission was effectively suppressed, as confirmed by the uniform dark contrast in a scan with the laser switched off (Figure 1b). 257 nm laser light at a power of 100 mW, measured before entering the entry window, was used to generate photoemission electron pulses. Every single pulse delivered energy of 20 nJ and, considering loss along the light path and electron optics, was converted to a pulsed electron probe containing about 100 e^−^/pulse. After passing through the condenser lens and the objective lens, pulsed electrons scanned the specimen with a dwell time of 64 µs/pixel in a 512 × 384 raster scan. Photoemission images were collected by averaging ten frames at each time delay to eliminate probable random noise. Spatial resolution loss was negligible, allowing the effective capture of spatial information in this study (Figure 1c).

### 2.2. Materials and Methods

The SAGCM InGaAs/InP APD consisted of a separated structure with an InP multiplication layer and an InGaAs absorption layer. At the interface between the absorption and multiplication layers, a layer of InGaAsP with a compositional gradient and a charge layer of InP were sandwiched. The top layer consisted of InGaAsP with a thickness of 300 nm, which absorbed most of the incident pump laser energy (515 nm). All the above-mentioned layers were deposited on an InP substrate and buffer layer. A cross-sectional view of the APD is shown in Supplementary Information Figure S1.

**Figure 2:**
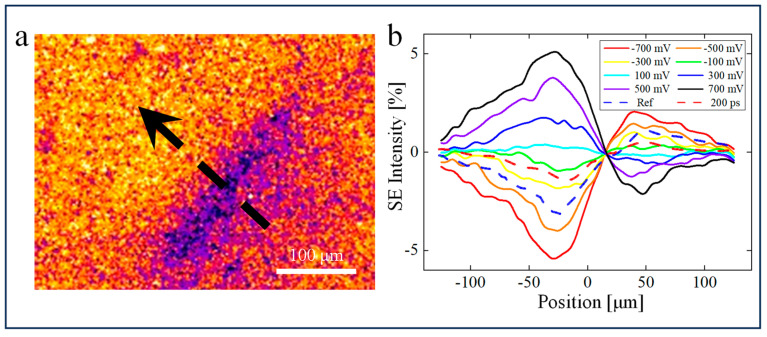
SPV-induced contrast on the surface of an APD. (**a**) Pump illumination induces a change in surface potential distribution, thereby forming a pronounced dark-to-bright contrast around the photoexcitation area. The arrow indicates the direction that the dipolar profile lies along and points to the ETD. (**b**) Simulated SE collection efficiency of different surface potentials and that found experimentally along the arrow. The Gaussian surface potential is set to diminish to 1/e^2^ of the maximum at −50 and 50 µm. Dashed lines show experimental cross-sectional profiles taken long before (labeled as Ref) and 200 ps after time zero. The SE collection efficiency is relative to the SE intensity far from the pump-excited area.

To correlate the contrast pattern induced by the pump laser to quasi-static surface potential and photogenerated carriers, we simulated the effect of surface potential on the trajectory of electrons. First, the electrostatic field in a three-dimensional model that largely replicated the internal conditions of the sample chamber was calculated. There is a Gaussian-distributed potential

(1) $$V\left(x,y\right)=V_{p}\exp\left[-\frac{1}{2}\left(\frac{x^{2}}{{\sigma_{1}}^{2}}+\frac{y^{2}}{{\sigma_{2}}^{2}}\right)\right]$$

on the sample surface. Corresponding to the scale of the pump radiation in the experiment, the width at 1/e^2^ of the maximum (*V_p_*) was 100 µm; that is, standard deviation $\sigma_{1}=25\;\mu m$ in Equation (1), where the *x*-axis was along the direction of the ETD (the arrow in Figure 2a). In the other axis direction, σ_2_ was set to 50 μm. Then, based on the above electrostatic simulation, we tracked the electrons escaping from a series of emission positions along the *x*-axis and counted the number of electrons collected by the ETD to obtain the SE collection efficiency. The details of the simulation are given in Supporting Information S2.

Since the static topographic features, chemical composition, and slow phenomenon arising from charged trap states make up the major contrast in photoemission images, it necessitates a reference-removal process to extract the contrast that reflects only the changes in local carrier density caused by optical excitation. Firstly, source drift caused by the delay line makes photoemission intensities change gradually over time. This can be illustrated by abstracting the brightness data for an area far away from the pumped area and arraying them as a function of time delay. To address this instability of photoemission, we normalized the dynamic images according to the intensity variation of the unpumped area. Furthermore, images captured hundreds of picoseconds before time zero where the system returns to equilibrium (called Ref) were subtracted to leave a homogeneous image in addition to the contrast that reflects a transient movie of charge carriers. The brightness and contrast parameters were kept the same for every frame throughout the image acquisition period.

## 3. Results and Discussion

In the thermionic emission mode, when the 515 nm pump laser (fluence ~0.15 mJ/cm^2^) was focused on the APD surface, an asymmetrical pattern characterized by a dark-contrast lobe (DCL) and bright-contrast lobe (BCL) appeared in the irradiated area (Figure 2a). Previous USEM results typically showed a symmetric elliptical bright (or dark) contrast with a profile similar to that of the pumping laser spot [35,36,37,38,39,40,41], which was attributed to the SE yield change caused by either the highly photoexcited electron energy [23,35] or the SPV effect [41,42,43,44]. The strikingly asymmetric USEM pattern we observed should originate from other factors with symmetry breaking. We noticed that the dipolar pattern lies along the direction pointing to the ETD, which indicates that it may be related to the variation in SE collection efficiency. Recently, similar dipolar USEM patterns have been observed for MAPbI_3_ [45], silicon [46], and GaAs [47]. As seen in these specific materials, SPV originating from trap states at the surface forms a local electric field that distorts the electric field above the sample surface and changes the trajectory (and thus collection efficiency) of SEs toward the ETD. It is noteworthy that this contrast pattern was the same as that obtained in photoemission mode at negative time delay before the arrival of the excitation pulses; that is, the Ref mentioned above. This can be understood by the observation that when the pump laser was turned off via a shutter, the pattern faded gradually within a few seconds. Therefore, this slow dissipation process dominates the average image in thermionic emission and the dark-to-bright contrast can be deemed constant compared to the periodic excitation of 5 MHz.

Microscopically, most of the photoexcited carriers (electron-hole pairs) generated by the super-bandgap illumination quickly recombine on the nanosecond time scale by a fast process in bulk. However, in the space charge region (SCR) near the surface, a certain portion of electron-hole pairs are separated, driven by the built-in electric field, and then pinned to the long-lived trap states with a lifetime much longer than the pulse interval (200 ns). After multiple laser pulses, carriers accumulate into the long-lived trap states, forming an elliptical charge distribution area that is consistent with the shape of the pumping laser spot on the sample surface, thus strengthening or compensating the intrinsic built-in electric field in the longitudinal direction. This Gaussian-distributed charge area and related electric field may affect the trajectory of SEs after escaping from the surface, resulting in different collection efficiencies of the SEs emitted from different areas as they are collected by the ETD.

To quantitatively demonstrate the process of dipolar pattern formation, we conducted a particle-tracing simulation of SEs. Figure 2b presents the simulation results of SE collection as a function of spatial position at different local surface potential strengths *V_p_* in the range from −700 to 700 mV in steps of 200 mV. For negative *V_p_*, the SE collection efficiency of positions near the ETD (i.e., on the right side of the curve) is higher. On the other side of the curve, electrons have a far lower probability of reaching the detector under the influence of the surface potential. The opposite behavior occurs for positive *V_p_*. Regardless of positive or negative potential, all the curves cross over a node around position X = 15 µm and change from bright to dark contrast or vice versa. For each curve, the minimum (maximum) value is located at X_1_ = −25 µm (−σ_1_) and the maximum (minimum) value at X_2_ = 50 µm (2σ_1_) for negative (positive) *V_p_*. The greater the *V_p_*, the greater the extreme values at these two points. In terms of the particle-tracing simulation in the space between the specimen surface and the detector, electrons generated near the ETD (BCL) are repelled to the ETD by the electric field arising with the negative *V_p_*. Conversely, the electrons generated at DCL move away from the detector, which leads to darker contrast. A similar nature of electron trajectory distortion works for positive *V_p_*, but appears in an opposite contrast array. The results for different potential widths are consistent with the above findings and the variation in the distance between extreme values Δ*X* with the surface potential distribution range has also been further verified (Supporting Information S2). Our simulation results confirm that the asymmetric dipolar contrast can indeed be attributed to the trapped carrier-induced SPV, which influences the trajectory and collection efficiency of low-energy SEs. Simultaneously, ultrafast photogenerated carrier motion can also be reflected in the contrast movie.

The experimentally observed cross-sectional SE collection efficiency along the same direction as in the particle-tracing simulation is also shown in Figure 2b. The pump laser creates a photovoltaic potential at the surface of about −400 mV [48]. The negative SPV means that it is electron carriers that are trapped at the surface states. The simulation results also show that the magnitude of the SPV is linearly related to the observed contrast magnitude, which means that the contrast changes originating from the SPV of different components can be directly superimposed. It is worth noting that the influence exerted by local potential on SE generation was not considered in the simulation.

**Figure 3:**
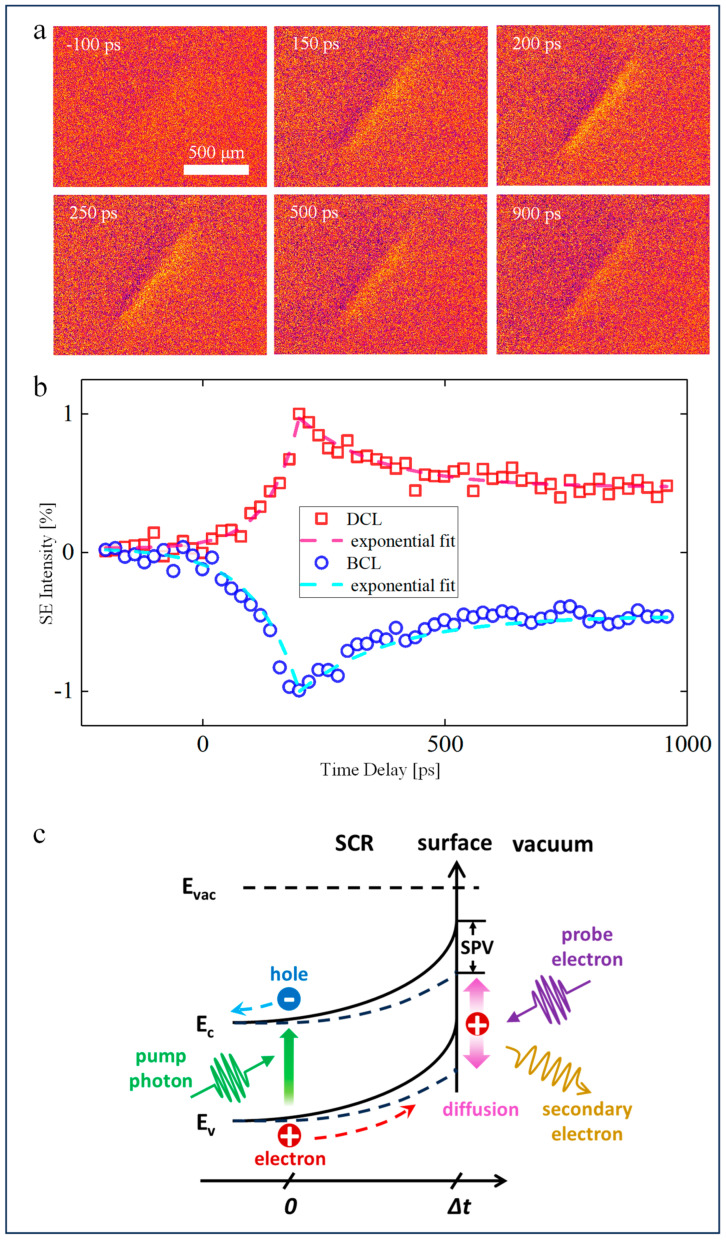
Time-resolved SE contrast variations of DCL and BCL and the energy band diagram illustrating contrast mechanisms. (**a**) Images obtained by background normalization and subtraction of the reference image acquired at far negative time. SPV-induced contrast is removed in ultrafast sequences, as can be seen from the uniform image at −100 ps, while the contrast change related to carrier lateral dynamics is left. (**b**) For both DCL and BCL, the maximum SE intensity variation reaches 200 ps after pump arrival and recovers subsequently with a critical time of about 170 ps fitted by exponential decay functions. (**c**) Schematic of excited carriers’ behavior under the surface photovoltage effect. The photogenerated carriers are attracted to the surface by the negative surface potential and then diffuse laterally. SPV, surface photovoltage; E_c_, conduction band bottom; E_v_, valence band top; E_vac_, vacuum level.

The contrast representing the transient behavior of optically excited carriers is pronounced in our USEM images, as shown in Figure 3, because the features arising from the static SPV of negative time delay are excluded from each snapshot USEM image by image processing, as mentioned above. The difference images also show a dipolar pattern but with opposite bright-to-dark contrast, indicating the imaging mechanism is the same as that of static USEM images. Following photoexcitation, the SE intensity of DCL increased monotonically with a time scale of τ_1_ ~80 ps (similar to that of heavily doped silicon of 58 ps for p-type and 77 ps for n-type [42]) determined by single exponential fitting, while that of BCL dimmed almost at the same time. After 200 ps, the lobes recovered gradually with a critical time of τ_2_ ~170 ps, indicating the recombination of photogenerated carriers. As seen below, the spatial contrast profiles broaden as the SE signal intensity decreases. Both lobes peak at a time delay of 200 ps, and considering that the absorption coefficient of InGaAsP is on the order of magnitude of 10^5^ cm^−1^ at 515 nm [49,50], a vertical drift velocity caused by an SPV of around 5 × 10^4^ cm/s is obtained.

As stated above, some electrons were consistently trapped at surface states under laser illumination with a high repetition rate, followed by a net charge redistribution within a thin SCR. As a result, a built-in electric field is formed in the SCR, while the bulk remains almost neutral. After each laser pulse, the newly excited electron-hole pairs are separated by the built-in field and drift in opposite directions. Specifically, the energetic hole carriers are driven toward the surface to partially neutralize the surface-trapped electron carriers (approximately 200 mV, shown as the red dashed line in Figure 2b), thus decreasing the surface potential and making the bipolar bright and dark contrast weaker. We attributed the slow rise time to the electron-hole separation below the surface under the built-in surface field. If the static SPV effect is subtracted, as we did, the transient surface potential modulated by photogenerated carriers results in the opposite dipolar contrast. Once the concentration of hole carriers on the surface reaches a critical threshold, the Coulomb repulsion takes over and suddenly widens the contrast distribution [42]. This process is shown schematically in Figure 3c.

**Figure 4:**
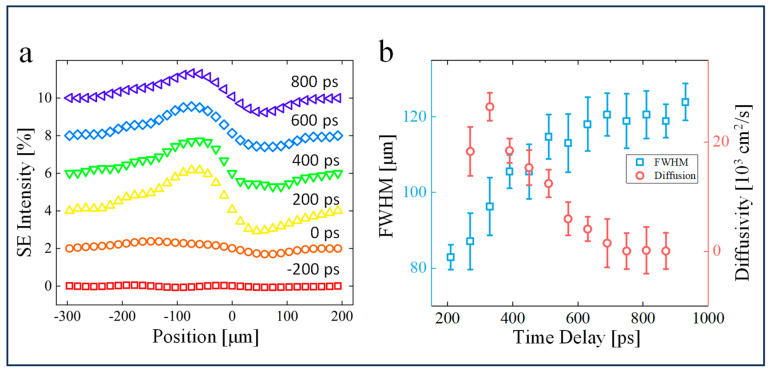
Surface diffusion of photogenerated carriers. (**a**) Diagonal cross-section of SE signals along the arrow in Figure 2a at different time delays. (**b**) Time evolution of the spatial carrier distribution and transient diffusivity. Error bars are the standard deviations obtained from different positions of the dipolar pattern.

Next, we consider the spatial diffusion dynamics of photoexcited carriers. Figure 4 shows the SE intensity distribution as a function of time delay, demonstrating the lateral expansion of photoexcited charge carriers on the APD surface. According to the quantitative analysis of the relationship between Δ*X* and the Gaussian potential carrier distribution mentioned above, the distance between extreme values and the full width at half maximum (*FWHM*) obeys the following relationship:

(2) $$FWHM=\frac{2}{3}\sqrt{2\ln2}∆X$$

From the relation between the diffusion coefficient *D(t)* and *FWHM* of the SE contrast [51],

(3) $$\frac{\partial {FWHM}^{2}(t)}{\partial t}=16\left(ln2\right)D(t)$$

we estimate that the lateral diffusion of the carrier population reaches a maximum of 2.5 × 10^4^ cm^2^/s at about 300 ps after pump excitation and then decreases exponentially with a time constant τ_d_ = 150 ps to less than 100 cm^2^/s at the largest time delay (900 ps) of our observation window. According to the Einstein relation

(4) $$\frac{D}{\mu }=\frac{kT}{e}$$

where *k* is the Boltzmann constant, *e* is the elementary positive charge, and *μ* is the hole mobility. The temperature of hole carriers reaches 10^5^ K because of photoexcitation and then drops to the lattice temperature upon carrier recombination. Note that we neglected the temperature dependence of the mobility [52]. The extremely high carrier temperature reflects the non-equilibrium nature of the laser-excited system, where the temperature is not a well-defined parameter. The decrease in diffusivity is directly related to the decay of carrier temperature, describing the establishment of thermalization between hot carriers and the environment. Similar transient super-diffusive behavior, which has been reported previously [38], indicates a ballistic transport feature, where transport occurs without scattering.

Finally, we would like to further discuss the underlying mechanism of the USEM image contrast, which still remains unclear. Both the energy gain and loss of charge carriers upon optical excitation in the bulk [23,35] and the photovoltage effect on the surface [41,42,43,44] have been proposed to qualitatively explain the origin of bright or dark USEM contrast. The Monte Carlo method has also been used to quantitatively simulate the effects of photoexcited carriers and SPV on the emission efficiency of SEs under low-energy primary electrons [44], but the influence of SPV on the trajectory and collection efficiency of Ses has not been considered. Our results add another important factor to consider for SE contrast. The actual contrast may be caused by the combined effects of the SE emission yield (consistent with the spot shape) and the external field on the SE collection efficiency (asymmetric distribution). In fact, several asymmetric USEM contrast images have been presented in previous reports [53,54], but the origin of the asymmetry was not discussed elaborately. The asymmetric pattern may be related to the strong SPV caused by specific materials and/or depend on the surface quality. Other factors affecting USEM image contrast may include the distribution of electric fields in the sample chambers of different USEM setups and the SE collection efficiency of ETDs. Further experimental and theoretical investigation is needed to quantitatively understand and clarify the mechanisms affecting USEM image contrast.

## 4. Conclusions

In summary, the spatiotemporal dynamics of surface carriers upon photoexcitation of an InGaAs/InP APD were closely examined in space and time using a home-built USEM. Theoretical simulations of electron tracing quantitatively confirmed the presence of image contrast arising from a −400 mV SPV potential, which can be visualized by conventional SEM observations. In addition to the generation and recombination, our time-resolved SEM movie also revealed the separation and drift of photoexcited carriers under the SPV effect in the time domain. From the observations of temporal and spatial contrast changes, the lateral diffusion coefficient of hot carriers reaches a maximum value of 2.5 × 10^4^ cm/s^2^ at 300 ps and then gradually decreases, demonstrating a transient super-diffusive behavior originating from the high kinetic energy of excited carriers and their equilibration with the environment. USEM provides a nascent technique for the characterization of surface processes initiated by the excitation of laser pulses that occur on ultrashort time scales and in the nanometer regime. It is expected that the continued development of USEM and new analysis techniques with high spatiotemporal resolution could pave the way to a profound understanding of nonequilibrium carrier mechanisms of semiconductor devices on ultrafast time scales.

## Data Availability

The data that support the findings of this study are available from the corresponding author upon reasonable request.

## Supplementary Materials

The following supporting information can be downloaded at https://www.mdpi.com/article/10.3390/nano14030310/s1, Figure S1: Schematic of the cross section of the planar separated absorption, grading, charge, and multiplication InGaAs/InP avalanche photodiode used in this study. To clarify all the layers, layers are not drawn to scale; Figure S2: Simulation of the electrostatic field in the specimen chamber using COMSOL. (a) Infrared photograph of the interior of the specimen chamber. The Faraday cage is in the upper left and the specimen stage is in the middle under the pole piece. (b) Simulated electric field distribution from which particle tracing was conducted. Color bars indicate component voltage; Figure S3: Simulated SE collection efficiencies of local Gaussian potentials with different widths. All percentages are relative to SEs collected without the surface potential. The simulation was conducted using a surface potential of −400 mV.
