# Supplementary material for: Spatiotemporal Visualization of Photogenerated Carriers on an Avalanche Photodiode Surface Using Ultrafast Scanning Electron Microscopy
Source: Nanomaterials (Basel). 2024 Feb 3;14(3):310. doi: 10.3390/nano14030310 (PMC10857202; doi:10.3390/nano14030310)
Supplement: Supplementary file 1 [file nanomaterials-14-00310-s001.zip › nanomaterials-2810911-supplementary.pdf]

Supplementary Materials

Spatiotemporal Visualization of Photogenerated Carriers on an Avalanche Photodiode Surface Using Ultrafast Scanning Electron Microscopy

Yuan Tian [1,2], Dong Yang [1,2], Yu Ma [1,2], Zhongwen Li [1], Jun Li [1], Zhen Deng [1], Huanfang Tian [1], Huaixin Yang [1,2], Shuaishuai Sun [1,3] and Jianqi Li [1,2,3,*]

[1] Beijing National Laboratory for Condensed Matter Physics, Institute of Physics, Chinese Academy of Sciences, Beijing 100190, China; tyan@iphy.ac.cn (Y.T.); dyang@iphy.ac.cn (D.Y.); mayu@iphy.ac.cn (Y.M.); lizhongwen@iphy.ac.cn (Z.L.); junli@iphy.ac.cn (J.L.); zhen.deng@iphy.ac.cn (Z.D.); hftian@iphy.ac.cn (H.T.); hxyang@iphy.ac.cn (H.Y.); sss@iphy.ac.cn (S.S.)
[2] School of Physical Sciences, University of Chinese Academy of Sciences, Beijing 100190, China
[3] Songshan Lake Materials Laboratory, Dongguan 523808, China
* Correspondence: ljq@iphy.ac.cn

S1 InGaAs/InP avalanche photodiode (APD)

We used an InGaAs/InP APD epitaxial material with the structure shown in Figure S1. The APD was grown by molecular beam epitaxy [1] and used without modification. The sample was cleaved and immediately transferred into the ultrafast scanning electron microscopy (USEM) specimen chamber under a vacuum of 1.6×10^{-4} Pa. According to the result of energy-dispersive spectroscopy mapping using transmission electron microscopy, the thickness of the top InGaAsP contact layer was 300 nm, which means that most of the pump light with a wavelength of 515 nm was absorbed by this layer.

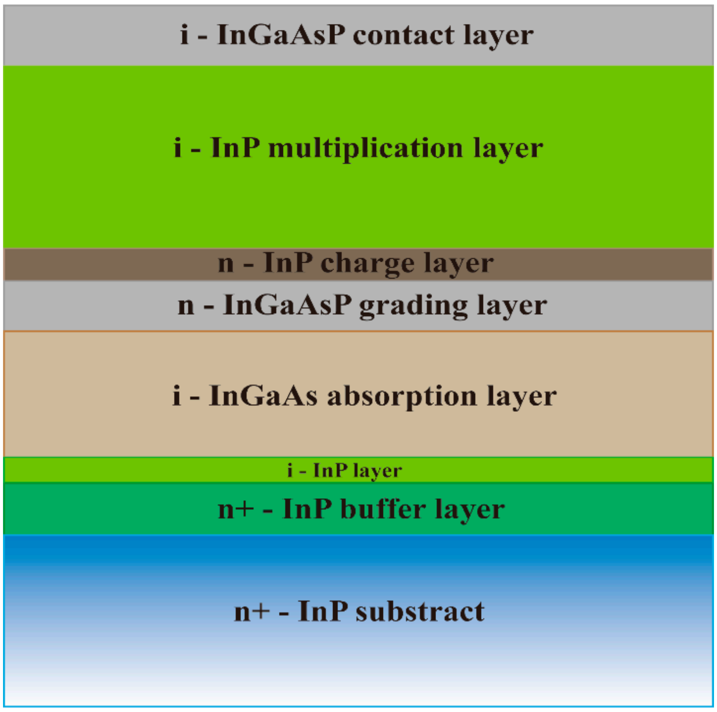

Figure S1. Schematic of the cross section of the planar separated absorption, grading, charge, and multiplication InGaAs/InP avalanche photodiode used in this study. To clarify all the layers, layers are not drawn to scale.

S2 Electron collection simulations

The simulations in this work were conducted using COMSOL Multiphysics. First, we constructed a 3D model of the interior of the specimen chamber of our USEM setup (Figure S2(a)) using the software. By the electrostatic interface, electric fields were computed with the boundary conditions that the pole piece and specimen stage were grounded and a positive bias voltage of 300 V was applied to the Faraday cage, consistent with the settings of our microscope. To study the role played by surface photovoltage, a 2D Gaussian potential distribution was generated on the surface of the specimen. The resulting electrostatic field is shown in Figure S2(b). Afterwards, based on this stationary solution, electrons were traced in this field with charged particle tracing interface and the electron collection efficiency was calculated by counting the number of electrons that reached the detector. The initial electron velocity was set at 5 eV with a direction following the Lambert cosine law, which is the typical kinetic energy of escaping secondary electrons (SEs). For comparison with the experimentally collected electron intensity profile, we performed a parametric sweep of the electron release position. The contribution of SEs generated by backscattered electrons to the total SE signal was ignored.

Figure S3 shows collection efficiencies of SEs emitted from positions along the x-axis with different potential ranges. The distance between the maximum and minimum positions, or peak-to-trough spacing, was essentially three times the standard deviation of the Gaussian potential in the x-direction; namely, $\Delta X = 3\sigma_1$. From this, the surface potential redistribution induced by transient lateral carrier diffusion was obtained. When the spatial inhomogeneity of excited electrons was considered, a profile different from that of the dipolar pattern could be found. In addition, potential distribution and internal configuration also affected the collection efficiency and final contrast formation [2, 3].

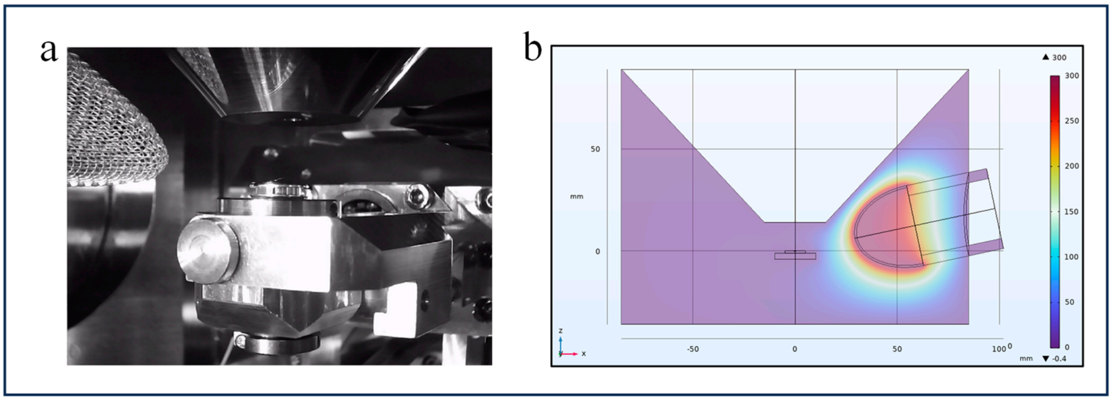

Figure S2. Simulation of the electrostatic field in the specimen chamber using COMSOL. (**a**) Infrared photograph of the interior of the specimen chamber. The Faraday cage is in the upper left and the specimen stage is in the middle under the pole piece. (**b**) Simulated electric field distribution from which particle tracing was conducted. Color bars indicate component voltage.

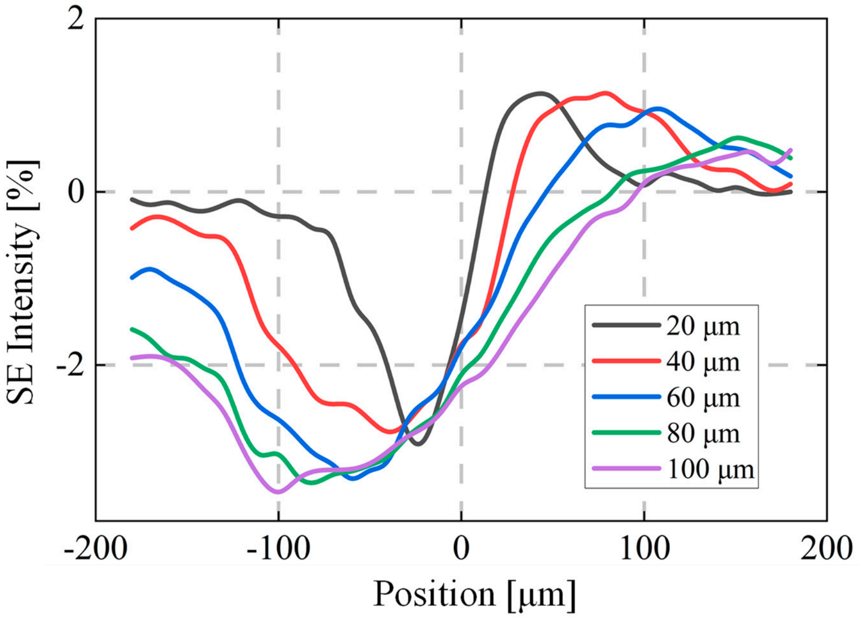

Figure S3. Simulated SE collection efficiencies of local Gaussian potentials with different widths. All percentages are relative to SEs collected without the surface potential. The simulation was conducted using a surface potential of −400 mV.

References

1. Zhang, J.; Li, X.; Du, C.; Jiang, Y.; Ma, Z.; Chen, H.; Jia, H.; Wang, W.; Deng, Z., Experimental Demonstration of the Impact of the Parameters of Floating Guard Ring on Planar InP/InGaAs-Based Avalanche Photodiodes' Performance and Its Optimization. *IEEE Photonics Journal* **2022,** 14, (2), 1-6.
2. Garming, M. W. H.; Bolhuis, M.; Conesa-Boj, S.; Kruit, P.; Hoogenboom, J. P., Lock-in Ultrafast Electron Microscopy Simultaneously Visualizes Carrier Recombination and Interface-Mediated Trapping. *The Journal of Physical Chemistry Letters* **2020,** 11, (20), 8880-8886.
3. Irde, G.; Pietralunga, S. M.; Sala, V.; Zani, M.; Ball, J. M.; Barker, A. J.; Petrozza, A.; Lanzani, G.; Tagliaferri, A., Imaging photoinduced surface potentials on hybrid perovskites by real-time Scanning Electron Microscopy. *Micron* **2019,** 121, 53-65.
